# Intrinsic foot joints adapt a stabilized-resistive configuration during the stance phase

**DOI:** 10.1186/s13047-020-0381-7

**Published:** 2020-03-12

**Authors:** Paul-André Deleu, Laurence Chèze, Raphaël Dumas, Jean-Luc Besse, Thibaut Leemrijse, Bernhard Devos Bevernage, Ivan Birch, Alexandre Naaim

**Affiliations:** 1 Univ Lyon, Université Claude Bernard Lyon 1, Univ Gustave Eiffel, IFSTTAR, LBMC UMR_T9406, F69622 Lyon, France; 2 Foot & Ankle Institute, Brussels, Belgium; 3 Hospices Civils de Lyon, Centre Hospitalier Lyon-Sud, Service de Chirurgie Orthopédique et Traumatologique, Lyon, France; 4 Sheffield Teaching Hospitals NHS Foundation Trust, Woodhouse Clinic, 3 Skelton Lane, Sheffield, S13 7LY UK

**Keywords:** Foot kinetics, Multi-segment foot, Inverse dynamics, Walking

## Abstract

**Background:**

This study evaluated the 3D angle between the joint moment and the joint angular velocity vectors at the intrinsic foot joints, and investigated if these joints are predominantly driven or stabilized during gait.

**Methods:**

The participants were 20 asymptomatic subjects. A four-segment kinetic foot model was used to calculate and estimate intrinsic foot joint moments, powers and angular velocities during gait. 3D angles between the joint moment and the joint angular velocity vectors were calculated for the intrinsic foot joints defined as follows: ankle joint motion described between the foot and the shank for the one-segment foot model (hereafter referred as Ankle), and between the calcaneus and the shank for the multi-segment foot model (hereafter referred as Shank-Calcaneus); joint motion described between calcaneus and midfoot segments (hereafter referred as Chopart joint); joint motion described between midfoot and metatarsus segments (hereafter referred as Lisfranc joint); joint motion described between first phalanx and first metatarsal (hereafter referred as First Metatarso-Phalangeal joint). When the vectors were approximately aligned, the moment was considered to result in propulsion (3D angle <60^o^) or resistance (3D angle >120^o^) at the joint. When the vectors are approximately orthogonal (3D angle close to 90°), the moment was considered to stabilize the joint.

**Results:**

The results showed that the four intrinsic joints of the foot are never fully propelling, resisting or being stabilized, but are instead subject to a combination of stabilization with propulsion or resistance during the majority of the stance phase of gait. However, the results also show that during pre-swing all four the joints are subject to moments that result purely in propulsion. At heel off, the propulsive configuration appears for the Lisfranc joint first at terminal stance, then for the other foot joints at pre-swing in the following order: Ankle, Chopart joint and First Metatarso-Phalangeal joint.

**Conclusions:**

Intrinsic foot joints adopt a stabilized-resistive configuration during the majority of the stance phase, with the exception of pre-swing during which all joints were found to adopt a propulsive configuration. The notion of stabilization, resistance and propulsion should be further investigated in subjects with foot and ankle disorders.

## Background

Adequate measurement of the intrinsic movement of the foot and ankle complex during walking has been impeded for decades by the simplified representation of foot as a single functional segment [1]. The development of three-dimensional (3D) multi-segment foot models partially tackled this major shortcoming of the established 3D lower limb models and showed their clinical value through the detection of intrinsic foot mobility impairments [1]. During the last decade, foot and ankle biomechanics were essentially described through the kinematics of the gait cycle as determined from cadaver, invasive bone pins, biplanar videoradiography and non-invasive surface marker studies, and plantar pressure measurements [2–8]. Recently, multi-segment kinetic foot models have received increasing attention in methodological and clinical studies providing new insights into how the intrinsic joints of the foot can have individual power distributions [9–12]. While kinematic multi-segment foot models can demonstrate the motion of the various intrinsic joints of the foot, establishing the kinetics of these joints represent a new series of challenges: definition of inter-segment joint centers, estimation of segmental shear forces and definition of segment inertial properties [11]. Despite these technical and methodological challenges, joint moments and powers have been able to provide new insights into the dynamic contribution of the Chopart and Lisfranc joints during gait, and new mechanisms of foot dysfunction in specific foot and ankle pathologies [11, 13, 14]. The Chopart joint has been described as the inter-segmental joint between the calcaneus and the midfoot segments whereas the Lisfranc joint was defined as the inter-segmental joint between the midfoot and the forefoot segments [4].

Based on the literature, kinetic analysis of intrinsic foot joints seems to be a valuable way for uncovering the role of foot and ankle during locomotion. However, the clinical interpretation of joint power remains an area of debate and not without controversies in the field of biomechanics. Although subject to challenge, joint power has been reported separately for the frontal, sagittal and transverse planes, which has revealed inconsistent results at the ankle [15–17]. The scientific community has also associated joint power with muscle action and energy transfer which has been widely criticized in the literature [18–20]. The difficulty is largely in the attribution of energy transfer (e.g. storage in elastic structures, muscle action) and in the allocation of forces to the agonist-antagonist and multi-joint muscles [10, 20]. The nature of the foot and ankle further increases the complexity of interpretation by the fact that, compared to the other major joints of the lower limb, intrinsic foot joints share common ligament and muscle tendon structures. Further analysis integrating in-vivo medical imaging [21] with musculo-skeletal models [22] or biplanar videoradiography [6] would be required to shed light on the contribution of each of the anatomical structures to foot and ankle function. It is therefore proposed that the joint power be supplemented by an angle (α_Mω_) which encapsulates a 3D angular relationship between the joint moment (**M**) and the joint angular velocity (**ω**) vectors, in an attempt to translate kinetic data into a “simple” functional relationship expressed in an accessible format applicable to the lower limb joints (ankle, knee, hip) [20]. When the 3D vectors **M** and **ω** are aligned (0° or 180°), the moment results in propulsion or resistance. When the 3D vectors **M** and **ω** are orthogonal (90°), the moment stabilizes the joint [20]. The 3D angle *α*_Mω_ between the joint moment (**M**) and the joint angular velocity (**ω**) revealed that the ankle joint generally adopts a resistive configuration (at midstance) followed by a propulsive configuration (at pre-swing) in healthy adults.

Based on current knowledge on the estimation of foot joint kinetics, this study proposes to expand the calculation of *α*_Mω_ to a four-segment kinetic foot model. Our hypothesis is that intrinsic foot joints are only partially propelling, resisting or stabilized due to the complex contributions of intrinsic and extrinsic foot muscles, ligaments and multiple joint surfaces. Therefore, the objective of this study was to analyse α_Mω_ at the Chopart, Lisfranc and First Metatarso-Phalangeal joints during the stance phase of gait and to investigate if these joints are predominantly propelling, resisting or stabilized. In addition, the percentage of propulsive/resistive moment (P/R %) contributing to drive each foot joint was also calculated. Angle *α*_Mω_ and P/R% were computed at the ankle joint with the foot considered to be a multi-segment system and a single segment for comparison.

## Methods

### Subjects

Twenty asymptomatic adult subjects participated in the study (male/female ratio 14/6; age (mean ± SD): 45.35 ± 11.97 years; height (mean ± SD), 1.75 ± 0.08 m; weight (mean ± SD): 75.5 ± 9.13 kg; BMI (mean ± SD): 24.62 ± 2.50 kg/m^2^; walking speed (mean ± SD): 1.39 ± 0.15 m/s). Participants were included if 1) they were able to walk barefooted independently, without support, 2) they had no history of orthopaedic, neurological or musculoskeletal problems affecting their gait. All participants were volunteers and signed the informed consent approved by the local ethical committee (B200–2017-061).

### Protocol

The simultaneous assessment of kinematics, kinetics, and plantar pressure measurements of each subject was achieved through the use of an advanced clinical examination platform combining a motion capture system, a force plate and a plantar pressure plate. The motion capture system consisted of 8 Miqus cameras (Qualysis, Göteborg Sweden) to capture the kinematic data (200 Hz) of the participant while walking over a 10 m walkway at a self-selected speed [23]. In the middle of the walkway, a Footscan® pressure plate (dimensions 0.5 m × 0.4 m, 4096 sensors, 2.8 sensors per cm^2^, RSscan International, Paal, Belgium) was mounted upon a custom made AMTI-force plate (dimensions 0.5 × 0.4 m, Advanced Mechanical Technology, Inc., Watertown, MA, US). The force plate was custom-made to fit the surface dimensions of the plantar pressure plate. This set-up allowed for the detection of specific gait events as well as for a continuous calibration of the pressure plate with the force plate using a Footscan® 3D interface box (RSscan International, Paal, Belgium). Data from the pressure and force plates were measured at a sampling rate of 200 Hz. The integration and synchronization of the three different hardware devices was achieved through the use of a Miqus Sync unit interface (Qualysis, Göteborg Sweden).

Thirty-two 8 mm retro-reflective markers were always mounted for each subject by the same clinician over anatomical landmarks according to the Instituto Orthopedico Rizzoli 3D multi-segment foot model (RFM) [4]. The skin markers were mounted using double-sided adhesive tape. After marker placement, the participants were asked to walk barefoot, at a self-selected speed until five valid trials were recorded. A trial was considered valid when the following criteria were met: 1) walking speed had to remain relatively constant across all trials of a recording session, 2) no visual gait adjustment was made by the subject during a trial to aim at the pressure plate and 3) a clear contact of the entire foot of interest within the boundaries of the sensor matrix of the pressure plate [24]. All marker trajectories were computed by Qualysis Tracking Manager 2.16 (Qualysis, Göteborg Sweden).

### Data analysis

**Fig. 1:**
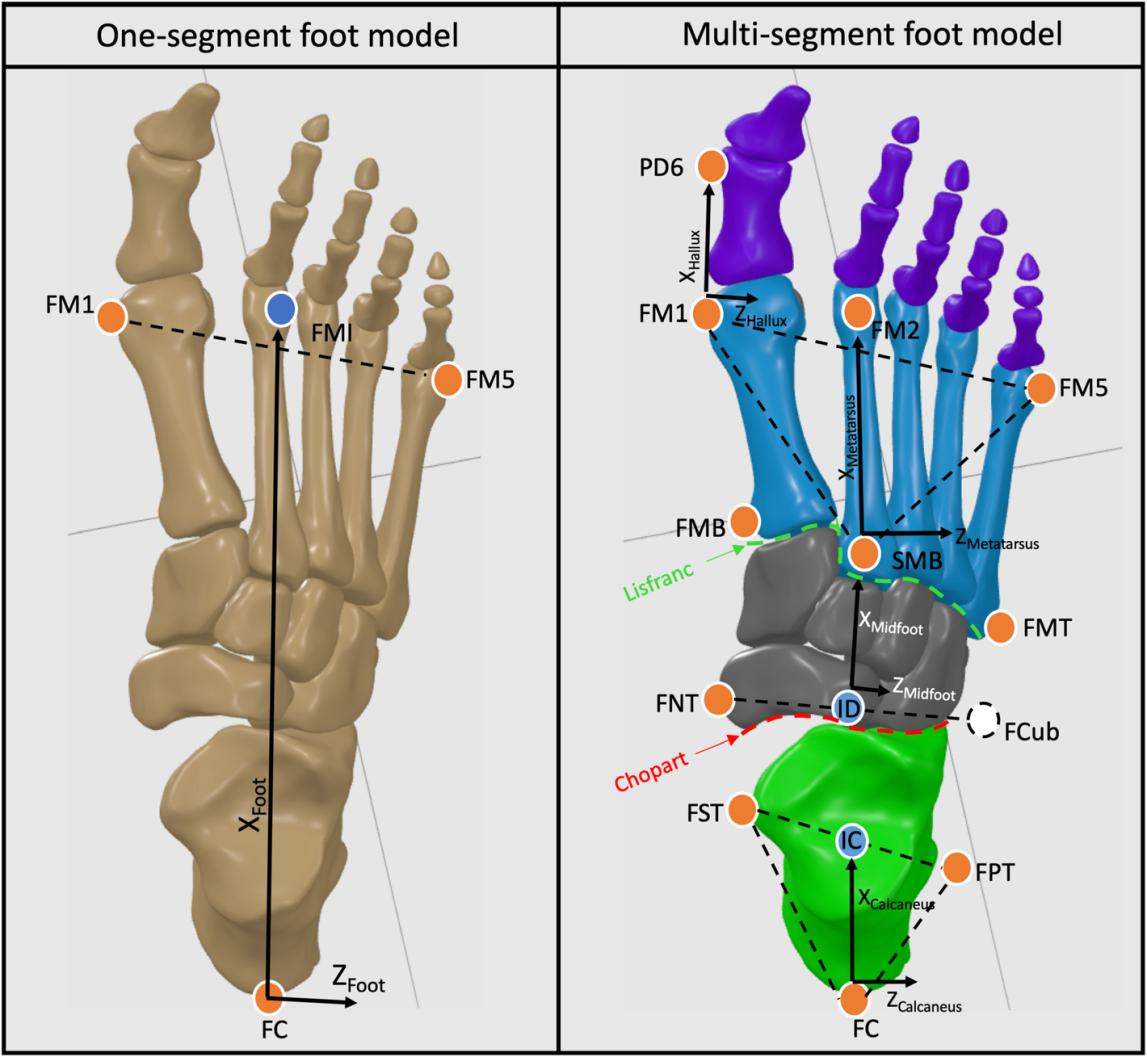
Inter-segment center definitions were defined according to an adapted version of Rizzoli foot model (Leardini et al. 2007) developed by Deschamps et al. (2017) (IOR-4Segment-model 1). Markers name: upper ridge of the posterior surface of the calcaneus (FC); peroneal tubercle (FPT); sustentaculum tali (FST); virtual cuboid marker (FCub), tuberosity of the navicular bone (FNT); first, second and fifth metatarsal base (FMB, SMB, FMT); first, second and fifth metatarsal head (FM1,FM2, FM5); PD6: distal dorso-medial aspect of the head of the proximal phalanx of the hallux; First Metatarso-Phalangeal joint center (FM1; Midfoot-Metatarsus center (SMB); Calcaneus-Midfoot center (ID)

Inter-segment 3D rotations were calculated according to an adapted version of Instituto Orthopedico Rizzoli 3D multi-segment foot model developed by Deschamps et al. (2017) (IOR-4Segment-model 1) following ISB recommendations, where dorsiflexion/plantarflexion (sagittal plane) is defined as rotation about the medio-lateral axis of the proximal segment, adduction/abduction (transversal plane) about the vertical axis of the distal segment and inversion/eversion (frontal plane) about an axis orthogonal to the first two axes (Fig. 1) [4, 25].

Joint forces (**F**) and moments (**M**) were computed in the Inertial Coordinate System by a bottom-up inverse dynamic method using a Newton-Euler recursive algorithm based on a homogeneous matrix formalism during the stance phase of gait [26]. Kinematic and force data were filtered using a low-pass zero-lag, 4th order, Butterworth filter, with a cut-off frequency of 10 Hz. Inertia and weight parameters of each foot segment were discounted as the inertia effects were negligible during gait compared to the external forces. The force plate data were distributed over each foot segment using the proportionality scheme described by Morlock & Nigg (1991) and validated by Saraswat et al. (2014) based on the distribution of the vertical ground reaction forces as measured by each sensor of the plantar pressure platform (i.e. if 15% of the total vertical force acted on the forefoot, it was assumed that 15% of the total horizontal force and vertical moment also acted on the forefoot) [13, 27]. The estimation of the subarea of each foot segment was achieved for each time frame by projecting the position of the retro-reflective markers vertically on the sensor matrix of the plantar pressure platform. The resulting center of pressure (CoP) of each estimated subarea was then used as the CoP for each foot segment in the inverse dynamics calculations. The joint moments were expressed in the proximal segment coordinate system.

For the computation of foot kinematics and kinetics, a virtual cuboid marker was created and defined as being at 2/3 of the distal distance between the peroneal tubercle and the base of the fifth metatarsal (Fig. 1). Inter-segment center definitions of the four segment foot model were based on Deschamps et al. (2017). For both kinematic and kinetic foot models, the ankle joint center was defined as the midpoint between the malleoli markers. Ankle joint motion was described between the foot and the shank for the one-segment foot model (hereafter referred as Ankle), and between the calcaneus and the shank for the multi-segment foot model (hereafter referred as Shank-Calcaneus) (Figure 1). Calcaneus-Midfoot (hereafter referred as Chopart joint) center was determined as being the midpoint between the cuboid and the navicular bone. Midfoot-Metatarsus (hereafter referred as Lisfranc joint) center was determined as being on the base of the second metatarsal. First Metatarso-Phalangeal joint center was the projection of first metatarsal head marker vertically at mid distance to the ground [11].

Joint power encapsulates the angular relationship between the **M** and the **ω** vectors and was computed according to the following equation:

$$ P=\left\Vert \mathbf{M}\right\Vert \times \left\Vert \upomega \right\Vert \times \cos {\upalpha}_{\mathrm{M}\upomega} $$

In supplement to the joint power, the *α*_Mω_ angle between the joint moment (**M**) and the joint angular velocity (**ω**) vectors was calculated as described by Dumas and Chèze (2008) following the present equation:

$$ {\upalpha}_{\mathrm{M}\upomega}={\tan}^{-1}\left(\frac{\left\Vert \mathbf{M}\times \upomega \right\Vert }{\mathbf{M}.\upomega\ }\right) $$

The *α*_Mω_ angle represents a positive value that ranges from 0 to 180°. Based on the *α*_Mω_ angle values, the kinetic behaviour of each joint was classified as followed:

- *Propulsion configuration* (**P**) corresponds to *α*_Mω_ ranging between 0 to 60° (*α*_Mω_ < 60°) where more than 50% of the 3D joint moment (i.e. cosα_Mω_ > 0.5) contributes to positive joint power.
- *Stabilization configuration* (**S**) corresponds to *α*_Mω_ ranging between 60 to 120° (*α*_Mω_ > 60° and < 120°) where less than 50% of the 3D joint moment (i.e. cosα_Mω_ < 0.5) contributes to joint power (positive or negative).
- *Resistance configuration* (**R**) corresponds to *α*_Mω_ ranging between 120 to 180° (*α*_Mω_ > 120°) where more than 50% of the 3D joint moment (i.e. cosα_Mω_ <  − 0.5) contributes to negative joint power.

The closer *α*_Mω_ is reaching 0 or 180°, the more the joint is almost driven by the joint moment (i.e. cosα_Mω_ → 1 *or* − 1) resulting in a maximized joint power [20]. In order to complementary illustrate this point, the percentage of propulsive/resistive moment (P/R % =100.cos(α_Mω_)) contributing to drive the joint is also given in Fig. 5. Inter-segment kinematic and kinetic computations were performed using an in-house constructed Matlab program. Joint moments and powers were normalized by subject-mass and all variables were time-normalized for the stance phase. The stance phase was separated on four phases (Table 1) based on the force and plantar pressure data.

**Table 1:** Subphases of the stance phase of gait

- **Loading Response:** The phase begins with initial floor contact and continues until the other foot is lifted for swing.	
- **Mid Stance:** It begins as the other foot is lifted and continues until body weight is aligned over the forefoot.	
- **Terminal Stance:** It begins with heel rise and continues until the other foot strikes the ground.	
- **Pre-Swing:** It begins with initial contact of the opposite limb and ends with ipsilateral toe-off.	

## Results

The 3D angle *α*_Mω_ and P/R % curves show that the four joints are never fully propelling, resisting or stabilized, but adopt a stabilized-resistive configuration during most of the stance phase, except at pre-swing with all joints in a propulsive configuration (Fig. 5). At loading response, all major joints quickly show a peak resistance (Ankle, Shank-Calcaneus, Lisfranc) or a stabilization configuration (Chopart) followed by a short period of stabilization occurring first at Ankle, Shank-Calcaneus and then for Lisfranc joints. The First Metatarso-Phalangeal joint demonstrates a propulsive configuration during loading response. During midstance, the Ankle and Shank-Calcaneus predominantly show a resistive configuration, whereas the Chopart adopts a stabilized-resistive configuration. In contrast, Lisfranc and First Metatarso-Phalangeal joints show a stabilized configuration. The propulsive configuration appears for Lisfranc joint first at terminal stance, then for other foot joints at pre-swing in the following order: Shank-Calcaneus, Ankle, Chopart and First Metatarso-Phalangeal joint.

### Ankle versus Shank-Calcaneus joints

The Ankle and Shank-Calcaneus joint powers remained low during the stance phase, except at loading response, when a peak of negative power occurred corresponding to a resistive configuration (both joints *α*_Mω_ ~ 161° & ~ 92% of resistive moment), and during pre-swing when a peak of positive power occurred corresponding to a propulsive configuration (Shank-Calcaneus ~ 67% of propulsive moment versus Ankle ~ 87% of propulsive moment) (Fig. 5). The *α*_Mω_ and P/R % of both joints demonstrated a high variability during loading response and at the end of midstance (Fig. 5).

**Fig. 2:**
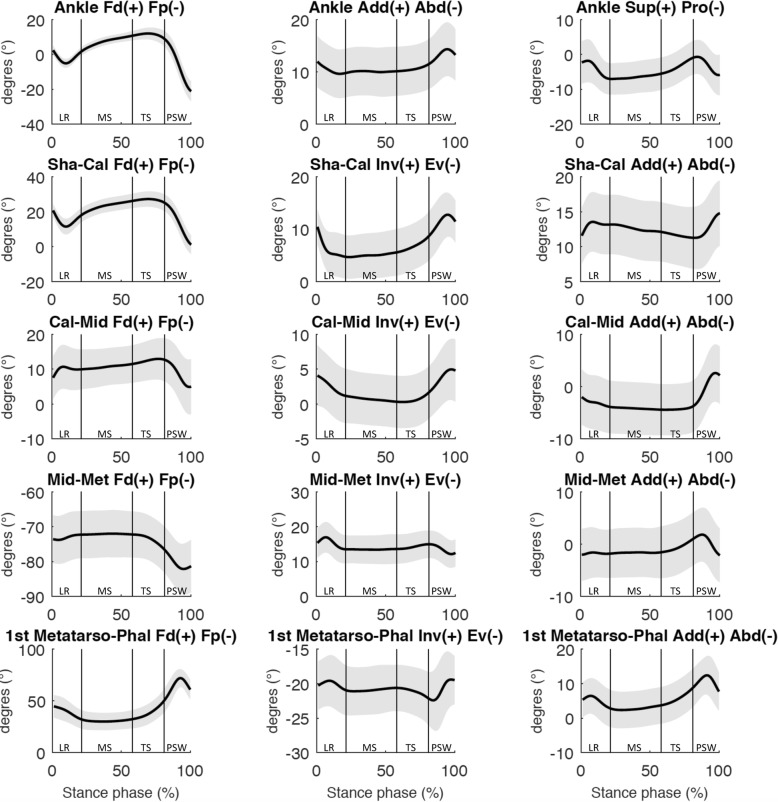
Mean 3D kinematics (degrees) for the Ankle, Shank-Calcaneus (Sha-Cal), Chopart joint (Cal-Mid), Lisfranc joint (Mid-Met), First Metatarso-Phalangeal joint (1st Metatarso-Phal). Standard deviations are visualized as bands. Abbreviations: LR: loading response; MS: midstance; TS: terminal stance; PSW: preswing phase. Each subphase of the stance phase of gait is delimited by vertical lines in each graph

**Fig. 3:**
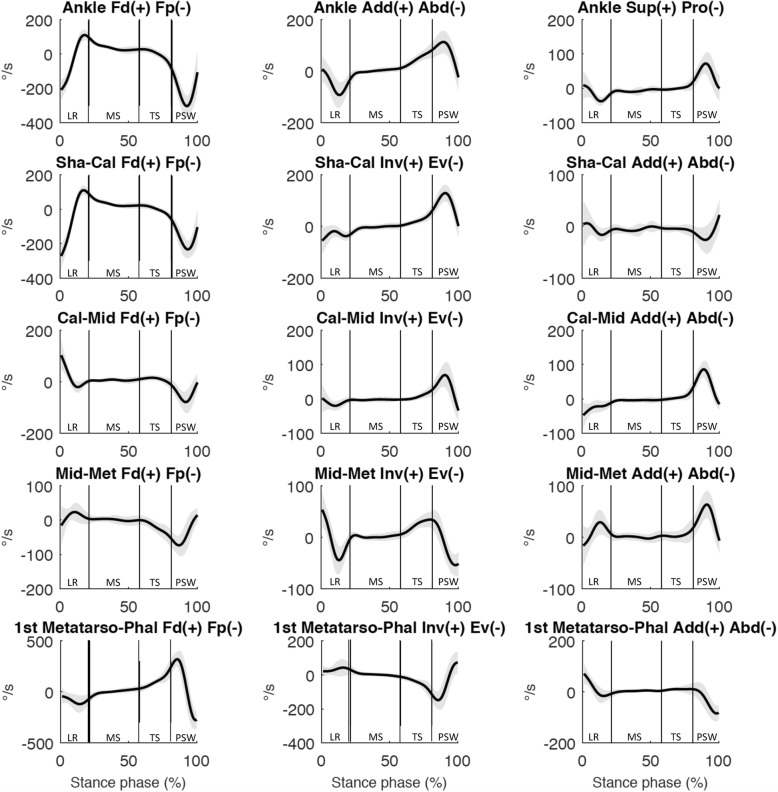
Mean 3D angular velocities (degrees/second) for the Ankle, Shank-Calcaneus (Sha-Cal), Chopart joint (Cal-Mid), Lisfranc joint (Mid-Met), First Metatarso-Phalangeal joint (1st Metatarso-Phal). Standard deviations are visualized as bands. Abbreviations: LR: loading response; MS: midstance; TS: terminal stance; PSW: preswing phase. Each subphase of the stance phase of gait is delimited by vertical lines in each graph

**Fig. 4:**
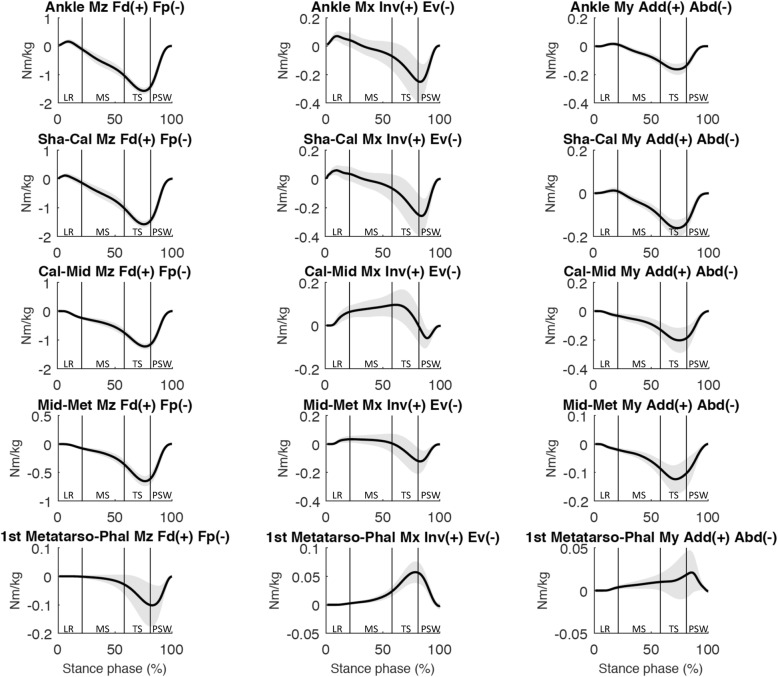
Mean 3D joint moments (Nm/kg) for the Ankle, Shank-Calcaneus (Sha-Cal), Chopart joint (Cal-Mid), Lisfranc joint (Mid-Met), First Metatarso-Phalangeal joint (1st Metatarso-Phal). Standard deviations are visualized as bands. Abbreviations: LR: loading response; MS: midstance; TS: terminal stance; PSW: preswing phase. Each subphase of the stance phase of gait is delimited by vertical lines in each graph

At loading response, the moments and angles (Fig. 2, 3 and 4) of both joints showed a predominantly dorsiflexion inter-segmental action, and a combination of plantarflexion and eversion movements. At midstance, the joint moments and angles of both joints showed a plantarflexion inter-segmental action and a dorsiflexion movement. At terminal stance and pre-swing, the joint moments and angles of both joints showed a predominantly plantarflexion inter-segmental action combined with a plantarflexion movement. Both peak power generation and absorption were lower in the Shank-Calcaneus joint than in the Ankle joint.

### Calcaneus-Midfoot (Chopart)

**Fig. 5:**
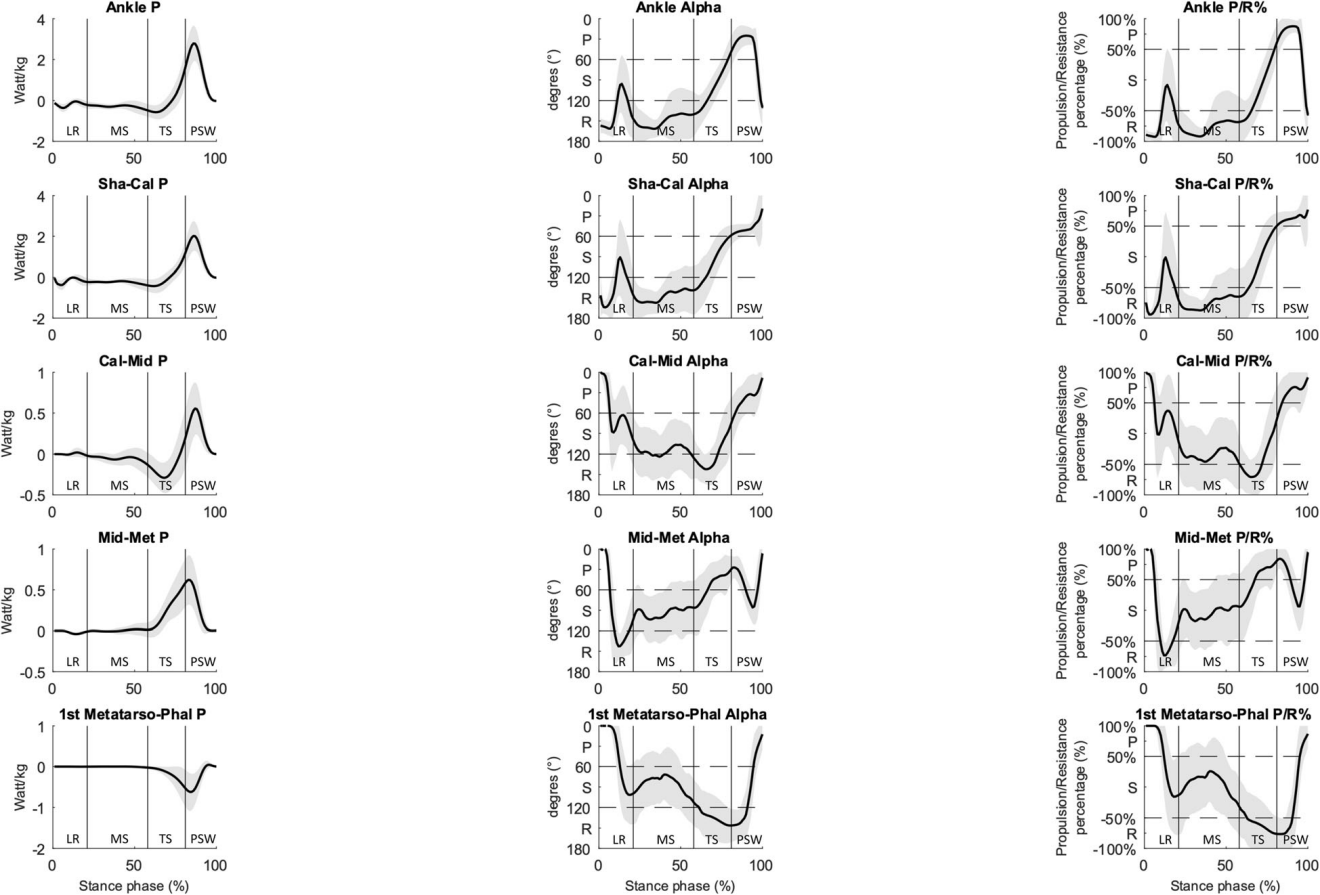
Mean 3D joint power (Watt/kg), mean α_Mω_ angle and mean percentage of propulsive/resistive moment for the Ankle, Shank-Calcaneus (Sha-Cal), Chopart joint (Cal-Mid), Lisfranc joint (Mid-Met), First Metatarso-Phalangeal joint (1st Metatarso-Phal). Standard deviations are visualized as bands. Subphases of the gait cycle. Abbreviations: R: resistance configuration; P: propulsion configuration; S: stabilisation configuration, LR: loading response; MS: midstance; TS: terminal stance; PSW: preswing phase. Each subphase of the stance phase of gait is delimited by vertical lines in each graph

The Calcaneus-Midfoot power remained low during the stance phase, except during terminal stance when a peak of negative power occurred corresponding to a resistive configuration (*α*_Mω_ ~ 143° & ~ 70% of resistive moment), and during pre-swing when a peak of positive power occurred corresponding to a propulsive configuration (*α*_Mω_ ~ 36° & ~ 75% of propulsive moment). The *α*_Mω_ and P/R % demonstrated a high variability during loading response and midstance (Fig. 5).

At loading response, Calcaneus-Midfoot power was negligible and the moments and angles showed a predominantly plantarflexion inter-segmental action and a combination of dorsiflexion and eversion movements (Fig. 2-4). Calcaneus-Midfoot power was also low during midstance and the *α*_Mω_ demonstrated a stabilized-resistive configuration. At terminal stance, the moments and angles showed a predominantly plantarflexion inter-segmental action combined with a dorsiflexion movement. At pre-swing, the moments and angles showed a predominantly plantarflexion inter-segmental action combined with a plantarflexion movement.

### Midfoot-Metatarsus (Lisfranc)

The Midfoot-Metatarsus power remained low during the stance phase, except at the end of terminal stance and the beginning of pre-swing when a peak of positive power was seen to occur corresponding to a propulsive configuration (*α*_Mω_ ~ 31°& ~ 78% of propulsive moment). The *α*_Mω_ and P/R % demonstrated a high variability during midstance and terminal stance (Fig. 5).

At loading response, Midfoot-Metatarsus power was negligible and the moments and angles showed a predominantly plantarflexion inter-segmental action and a combination of dorsiflexion and inversion/eversion movements (Fig. 2-4). Midfoot-Metatarsus power were also low during midstance and the *α*_Mω_ demonstrated a stabilized configuration (~ 90°). At terminal stance, the moments and angles showed a predominantly plantarflexion inter-segmental action combined with a plantarflexion movement. The moments and angles at the transition between terminal stance and pre-swing showed a predominantly plantarflexion inter-segmental action combined with a plantarflexion movement. In contrast to the Ankle and Chopart joints, the Lisfranc joint demonstrated a stabilized configuration at the end of pre-swing. The moments and angles showed an eversion inter-segmental action combined with an eversion movement.

### First Metatarso-Phalangeal

The First Metatarso-Phalangeal power remained low during the stance phase, except at pre-swing when a peak of negative power was seen to occur corresponding to a resistive configuration (peak at *α*_Mω_ ~ 146° & ~ 76% of resistive moment). The *α*_Mω_ and P/R % demonstrated a high variability during the entire stance phase, except during pre-swing (Fig. 5).

The First Metatarso-Phalangeal power was negligible from loading response to terminal stance. 3D angle *α*_Mω_ and P/R % showed a propulsive configuration at loading response and a stabilized configuration during midstance. At terminal stance and pre-swing, the moments and angles showed a predominantly plantarflexion inter-segmental action combined with a dorsiflexion movement (Fig. 2-4).

## Discussion

The current study proposes the use of the *α*_Mω_, which encapsulates a 3D angular relationship between the joint moment (**M**) and the joint angular velocity (**ω**) vectors, in an attempt to provide a “simple” measure of the function of intrinsic foot joints during gait. Our hypothesis was confirmed by the results which showed that the intrinsic foot joints are never fully propelling, resisting or stabilized, but instead adopt a stabilized-resistive configuration during most of the stance phase, with the exception of during pre-swing when all joints adopt a propulsive configuration. This stabilized-resistive configuration keeps the foot from collapsing while bearing weight, allowing stabilization of the foot and thus accomplishing the stability requirements of locomotion [28].

This study expanded the calculation of α_Mω_ from a lower limb model to a four-segment kinetic foot model. The **α**_**Mω**_ pattern of the Ankle joint found in this study was generally similar to that proposed by Dumas and Chèze (2008) [20]. The most notable difference between the results of the two studies was that during loading response Dumas and Chèze (2008) found a stabilized configuration as opposed to the resistive configuration found in this study. The decomposition of α_Mω_ revealed that this discordance in configuration is likely to arise from different kinematic patterns, as Dumas and Chèze (2008) found a predominant combination of abduction and external rotation movements, whereas this study showed a combination of plantarflexion and eversion movements. It may be concluded that the observed differences may therefore come from the variation in foot kinematics between participants, since both studies used the same joint center, anatomical landmarks and reference frame to model the ankle joint.

A point of interest which deserves discussion is the critical role of the method by which the ankle complex is modelled. The simplified representation of the foot as a single functional segment is still widely used to quantify ankle joint kinetics in clinical biomechanical studies. The results showed that both peak power generation and absorption were lower in the Shank-Calcaneus joint than in the Ankle joint (Fig. 5). This is in accordance with previous gait studies for asympatomatic [29–31] and symptomatic [32] subjects. However, in terms of α_Mω_ and P/R% waveforms, the Shank-Calcaneus joint and the Ankle joint showed similar waveforms during the stance phase of gait.

As *α*_Mω_ is simply an extension of the joint power, it not possible to directly relate the propulsion/resistance or stabilization configuration to a particular anatomical structure crossing the joint. For instance, a resistance configuration does not systematically reveal an eccentric action of the muscles but the tension of tendons, ligaments, fascias and skin. Still, *α*_Mω_ can be interpreted with regards to the foot functional anatomy. Adding α_Mω_ to the computation of foot kinetics creating a four-segment foot model enabled the discovery of new insights into how the Chopart and Lisfranc joints are contributing to foot function from midstance to pre-swing. However, the interpretation of *α*_Mω_ of both joints during loading response should be undertaken with care, as the forefoot may not yet be in contact with the ground, and their respective joint moments were found to be close to zero. The computed *α*_Mω_ of both joints appear to correspond with their respective functional anatomy. The Lisfranc joint shows predominantly a stabilized configuration during midstance, possibly caused by the anatomical stiffness of the tarsometatarsal joints. The passive stability of the Lisfranc joint is largely provided by the plantar ligaments and the second metatarsal with its encased base between the cuneiforms. The peroneus longus tendon, inserted at the plantar aspect of the first metatarsal base, and the first cuneiform further contribute to the stabilization of the first ray in opposition to dorsiflexion moments that are commonly exerted by ground reaction forces acting plantar to the first metatarsal head [33]. In contrast to the Lisfranc joint, the Chopart joint has considerably more freedom of movement and requires a resistive-stabilized configuration to control the deformation of the longitudinal arch under load, and to avoid collapsing during midstance and propulsion. Recent evidence suggests that the stability of the longitudinal arch is not only provided by the passive structures (e.g. plantar ligaments and plantar fascia), but also by contraction of the plantar intrinsic foot muscles [34]. These muscles act as local stabilizers increasing the inter-segmental stability of the longitudinal arch. They have small cross-sectional areas and therefore produce small rotational moments [34]. Flexor hallucis longus and tibialis posterior provide further substantial dynamic support to the medial longitudinal arch. These muscles provide both resistive and propulsive capabilities during gait [35, 36].

The foot’s rigidity in late stance is mainly attributed to the windlass and midtarsal locking mechanisms [37, 38]. The stiffening of the foot is required to resist the ground reaction forces and allow efficient propulsion of the body in late stance. At heel off, α_Mω_ and P&R % waveforms of the Ankle and Lisfranc joints are simultaneously adopting a propulsive configuration at terminal stance, which means that both joints are predominantly being driven by their respective plantarflexion moments, and thus contributing to power generation (Fig. 4-5). Recent studies suggest that this power generation at the Lisfranc joint during terminal stance is the result of the Windlass mechanism [11, 29, 39]. The activation of this mechanism results in tension the plantar fascia by winding it around the metatarsal heads as the toes dorsiflex in terminal stance [38]. The power generated at the Lisfranc joint would then in turn result in the optimal repositioning of the bones around the Chopart joint [40]. The reorientation of the midfoot bones were mainly characterized in our results by a plantarflexion moment combined with a dorsiflexion and inversion movement of the Chopart joint resulting in a resistive configuration. This phenomenon is often referred in the literature as the midtarsal locking mechanism [37, 41]. However, the term “locking” seems inappropriate as rotational movement at the Chopart joint was observed at terminal stance. It has also been suggested that the increased tension in the plantar fascia, and possibly other muscle-tendon structures, would result in a shortening and rise of the longitudinal arch through flexion and adduction of the metatarsals in combination with an inversion of the rearfoot [38, 42]. The longitudinal arch raise would then induce a first ray plantarflexion, an inversion of the Chopart joint, an inversion of the rearfoot, and Ankle dorsiflexion [40]. At 65% of the stance, the resistive configuration adopted by the Chopart joint is converted into a propulsive configuration where the moments and angles show predominantly a plantarflexion inter-segmental action combined with a plantarflexion movement (Fig. 2-5). This configuration conversion allows the Chopart joint to contribute to power generation. Elastic recoil of the tibialis posterior as well as of the plantarflexors of the ankle and toes’ further add to power generation at the Chopart and ankle joints during terminal stance and pre-swing [11].

A last point of interest is the functioning of the First Metatarso-phalangeal joint during propulsion, which tends to absorb relatively more power than the joints distal to the Ankle joint (Fig. 5). The Ankle and the First Metatarso-Phalangeal joints, among all joints of the foot, undergo the largest ranges of motion in the sagittal plane, while moving in opposite directions during the majority of the stance phase of gait. Both joints are crossed by the tendon of flexor hallucis longus, which acts as a plantarflexor of the ankle and a joint-stabilizer of the First Metatarso-Phalangeal joint. Further active stabilization of the hallux against the ground is provided by the flexor hallucis brevis, adductor and abductor hallux muscles which exert a plantar flexion moment. Evidence suggests that this power absorption observed at the First Metatarso-Phalangeal joint could be the result of the pressing down action of the intrinsic foot muscles and the flexor hallucis longus to stabilize the hallux against the ground and to counteract the dorsiflexion and eversion moments externally produced by the ground reaction forces [43, 44]. Kelly et al. (2014) further suggested that the intrinsic foot muscles also served to decrease the stress on passive elements, such as the plantar ligaments, plantar fascia and plantar plate, crossing the First Metatarso-Phalangeal joint [45]. It may therefore be concluded that the resistive configuration adopted by the First Metatarso-Phalangeal joint at terminal stance and pre-swing is in accordance with earlier findings describing the mechanisms countering the ground reaction forces.

There are several limitations to this study. A first issue concerns the estimation of the center of pressure and resultant ground reaction forces for each foot segment, derived from combining force and pressure data. The use of a proportionality scheme was originally validated for the calculation of joint kinetics of a three segment foot model and not for a four segment foot model [13]. Validity of the proportionally scheme was assessed by comparing the predicted shear forces obtained from the same experimental setup as the present study with the measured shear forces obtained by asking the participants to adopt a 3 step controlled foot placement approach on two adjacent force plates during a walking trial. Mean differences of less than 3% between the shear force measured by 2 adjacent force plates and the shear force predicted by the proportionality scheme in the hindfoot and forefoot segments were found in a paediatric population. Recently, Eerdekens et al. (2019) has further investigated the clinical applicability of the proportionality scheme in subjects suffering from ankle and hindfoot osteoarthritis [14]. Their results revealed insignificant over- and underestimation errors in multi-segment foot kinetics by comparing estimated shear forces with measured shear forces obtained by an adjacent force plate method. However, these results should be viewed with care as errors in the determination of the point of force application have been found towards force plate edges [46]. Therefore, the results of the current study should be considered as an estimation and further research is needed.

A second limitation is the use of skin markers to estimate joint centers and segmental kinematics. Estimation of movement of foot bones using skin markers, especially in complex joints such as the Chopart and Lisfranc joints, is a challenging process complicated by the small size of the foot bones as well as the relatively small motions occurring at these joints. Over the last decade, multi-segment kinematic foot models using skin markers have been proposed to estimate the kinematic behaviour of foot joints by grouping foot bones into segments (e.g. hindfoot, midfoot, forefoot), the clinical value of which has been shown through the detection of intrinsic foot mobility impairments [1, 47]. However, this approach can lead to inaccuracies in foot joint kinematic and kinetic estimations as these models do not account for individual bone-to-bone motion and therefore may violate rigid-body assumptions [2, 8]. In addition, soft-tissue artefacts must be considered in segmental foot analysis [2, 6, 8]. To overcome these challenges, methods using bone-anchored markers and biplanar videoradiography have been used to provide more accurate measures of foot joint motion, which can be difficult to discern with skin mounted markers [2, 6–8]. However, the invasive/ ionising nature of these alternative methods precludes their use in routine clinical analysis. To assess the errors in experimental data due to violation of the rigid-body assumption, studies have compared bone-mounted markers with skin-mounted markers [2, 8], which found no systematic error pattern in the degree of skin motion over the underlying foot bones. They also reported that the degree of error varied between subjects and between anatomical sites and found maximum differences of 3 to 9 degrees between skin and bone-mounted marker data [2, 8]. Recently, Kessler et al. (2019) compared foot motion measured by biplanar videoradiography and optical motion capture [6]. They found good agreement between the two systems for foot motion in the sagittal plane, and reported soft-tissue artefacts of 3.29 mm on the surface of the foot [6]. However, the impact of these errors on the estimation of foot joint moments, angular velocity and powers is difficult to assess. Therefore, the results of the current study should be considered as an estimate, and further research using emerging technologies such as biplanar videoradiography is needed to provide a more detailed insight into the kinetic behavior of foot joints.

A third limitation concerns the recruitment of asymptomatic participants, which does not mean that all feet were entirely free of degenerative changes in foot structure (e.g. clinical osteoarthritic changes). Studies have shown that a sizeable percentage of asymptomatic individuals may present abnormal findings of soft tissues on magnetic resonance imaging [48, 49]. Finally, since walking speed results in different foot kinetics, the effect of walking speed on *α*_Mω_ should be further investigated in future studies [11].

## Conclusion

This study reports a first attempt to gain additional insight into the kinetic behaviour of multiple foot joints through the use of a “simple” variable (*α*_Mω_) during gait. Intrinsic foot joints adopt a stabilized-resistive configuration during the majority of the stance phase. Results of the current study should be considered with care as skin markers and a proportionality scheme were used to estimate foot joint kinematics and kinetics. The notion of stabilization, resistance and propulsion should be further investigated in subjects with foot and ankle disorders.

## Data Availability

The datasets generated during the current study are available from the corresponding author on reasonable request.

## Abbreviations

3D: three-dimensional
BMI: body mass index
**M**: joint moment vector
P: propulsion configuration of the joint
P/R %: percentage of propulsive/resistive moment
R: resistance configuration of the joint
S: stabilization configuration of the joint
**ω**: joint angular velocity vector
